# A Systematic Map of Systematic Reviews in Pediatric Dentistry—What Do We Really Know?

**DOI:** 10.1371/journal.pone.0117537

**Published:** 2015-02-23

**Authors:** Ingegerd A. Mejàre, Gunilla Klingberg, Frida K. Mowafi, Christina Stecksén-Blicks, Svante H. A. Twetman, Sofia H. Tranæus

**Affiliations:** 1 Swedish Council on Health Technology Assessment, Stockholm, Sweden; 2 Department of Pediatric Dentistry, Faculty of Odontology, Malmö University, Malmö, Sweden; 3 Department of Odontology, Section for Pediatric Dentistry, Faculty of Medicine, Umeå University, Umeå, Sweden; 4 Department of Odontology, Section for Cariology, Endodontics, Pediatric Dentistry and Clinical Genetics, Faculty of Health and Medical Sciences, University of Copenhagen, Copenhagen, Denmark; UNC School of Dentistry, University of North Carolina-Chapel Hill, UNITED STATES

## Abstract

**Objectives:**

To identify, appraise and summarize existing knowledge and knowledge gaps in practice-relevant questions in pediatric dentistry.

**Methods:**

A systematic mapping of systematic reviews was undertaken for domains considered important in daily clinical practice. The literature search covered questions in the following domains: behavior management problems/dental anxiety; caries risk assessment and caries detection including radiographic technologies; prevention and non-operative treatment of caries in primary and young permanent teeth; operative treatment of caries in primary and young permanent teeth; prevention and treatment of periodontal disease; management of tooth developmental and mineralization disturbances; prevention and treatment of oral conditions in children with chronic diseases/developmental disturbances/obesity; diagnosis, prevention and treatment of dental erosion and tooth wear; treatment of traumatic injuries in primary and young permanent teeth and cost-effectiveness of these interventions. Abstracts and full text reviews were assessed independently by two reviewers and any differences were solved by consensus. AMSTAR was used to assess the risk of bias of each included systematic review. Reviews judged as having a low or moderate risk of bias were used to formulate existing knowledge and knowledge gaps.

**Results:**

Out of 81 systematic reviews meeting the inclusion criteria, 38 were judged to have a low or moderate risk of bias. Half of them concerned caries prevention. The quality of evidence was high for a caries-preventive effect of daily use of fluoride toothpaste and moderate for fissure sealing with resin-based materials. For the rest the quality of evidence for the effects of interventions was low or very low.

**Conclusion:**

There is an urgent need for primary clinical research of good quality in most clinically-relevant domains in pediatric dentistry.

## Introduction

To help insure that administered treatments do more good than harm, gaps in knowledge about their effects—uncertainties—must be identified, and those deemed sufficiently important must be addressed [1]. According to the Database of Uncertainties about the Effects of Treatments (DUETs) and the Swedish Council on Health Technology Assessment (SBU), a knowledge gap is present when systematic reviews reveal uncertainty about a health technology’s medical effects, or if no systematic literature review is available (http://www.library.nhs.uk/duets/), (http://www.sbu.se/en/Published/Scientific-Uncertainties/). It follows that systematic reviews based on high-quality studies are crucial, not only for assessing the best available evidence, but also for identifying and communicating scientific uncertainty (knowledge gaps). Besides offering practitioners and other decision-makers an overview, an important goal is to encourage clinical research in strategic areas linked to clinical management. An initial step in this process is to systematically and transparently describe the extent of research in a field and to identify gaps in the research base [2].

In 2010, the Swedish Government gave SBU the task of identifying knowledge gaps in health care. Summarizing the state of research knowledge in the field of pediatric dentistry was considered to be an important part of this assignment. Pediatric dentistry involves early diagnosis and treatment of the multitude of oral diseases and conditions found in the child’s and the adolescent’s mouth, including caries, periodontal disease, mineralization disturbances, disturbances in tooth development and tooth eruption, and traumatic injuries [3].

A number of systematic reviews addressing various topics in the field of pediatric dentistry have been published. However, to our knowledge their methodological quality has not been systematically assessed and the state of research knowledge of common interventions in pediatric dentistry has not been compiled. Using the mapping approach, the aim of this study was to identify, appraise and summarize existing knowledge and identify knowledge gaps covering essential fields of oral health care in children and adolescents. The mapping should provide answers to the most relevant questions related to pediatric dentistry. For example, since dental caries is the most common chronic disease among children and adolescents [4] it is crucial for the practitioner as well as the community to know which methods are most effective for preventing and treating the disease. Another example is dental anxiety/behavior management problems where the reported prevalence exceeds ten percent in many countries [3]. Knowledge about the best strategies for managing these children is obviously important. It is equally important to identify gaps in the research base so that unanswered questions can be tackled by additional practice-relevant research activities. For practical reasons the mapping was restricted to ten domains and did not include oral manifestations of malignant diseases, oral mucous lesions, surgery and orthodontics. AMSTAR [5] was used as the basis for assessing the quality of relevant systematic reviews.

## Material and Methods

After consulting specialists in pediatric dentistry and colleagues working in community dentistry, questions related to the following ten domains appeared to cover the most important activities in pedodontic clinical practice: behavior management problems/dental anxiety; caries risk assessment and caries detection, including radiographic technologies; prevention and non-operative treatment of caries in primary and young permanent teeth; operative treatment of caries in primary and young permanent teeth; prevention and treatment of periodontal disease; management of tooth developmental and mineralization disturbances; prevention and treatment of oral conditions in children with chronic, diseases/developmental disturbances/obesity; diagnosis, prevention and treatment of dental erosion and tooth wear; treatment of traumatic injuries in primary and young permanent teeth and cost-effectiveness of interventions.

### Inclusion criteria

Systematic reviews published in peer-reviewed journals addressing questions on any of the selected domains. Intervention, control and outcome parameters in accordance with the particular question:

**Table:** 

Population	Children and adolescents up to age 18
Intervention	Diagnostic testing, prediction, prevention, treatment
Control	Reference test, control (comparator)
Outcome	Accuracy, validity, effect of intervention, cost-effectiveness

### Exclusion criteria

- Surgical intervention of cleft lip and palate
- Speech-related interventions
- Guidelines or non-systematically performed meta-analyses

### Literature search strategy

**Fig 1:**
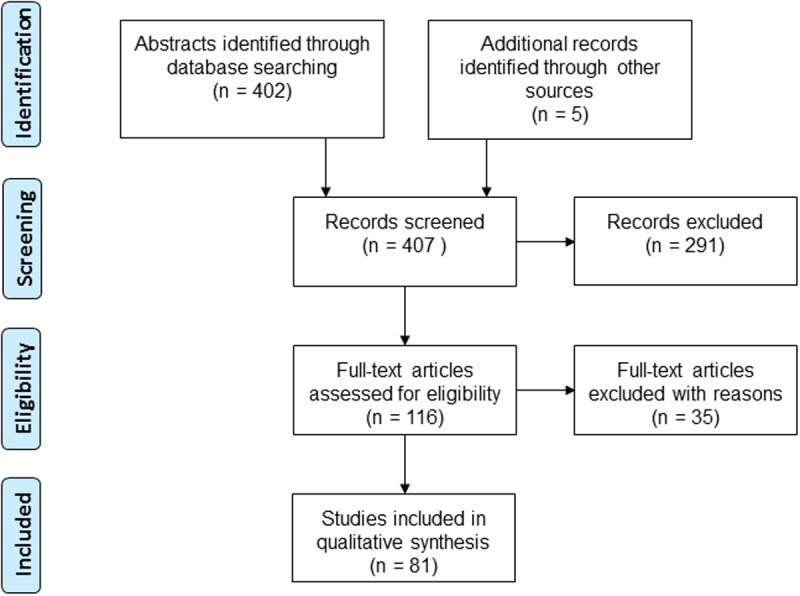
Flow diagram showing the literature search strategy. Flow diagram chart showing the literature search strategy with the number of retrieved abstracts, included and excluded articles.

The latest literature search was made in April 2014 in three databases: PubMed, The Cochrane Library and the Centre for Reviews and Dissemination (CDR). There were no language restrictions. The search algorithm was (“Child” [Mesh] OR children[tiab] OR “Adolescent”[Mesh] OR adolescent[tiab]) AND (“Dental Care”[Mesh] OR dental care[tiab] OR “Dental Caries”[Mesh] OR caries[tiab]) AND systematic[sb]. Screening of references was used. The numbers of retrieved abstracts, included and excluded articles at each stage of the search process are given in a flow diagram (Fig. 1). Abstracts identified according to the inclusion criteria were examined independently by two review authors. If at least one of them found an abstract potentially relevant, it was included and the article was ordered in full text.

### Data extraction and quality assessment

Data extraction, assessment of relevance and quality of included reviews were undertaken independently by two review authors. Any differences were solved by consensus; a third review author was consulted if necessary. In the case of reviews in which one of the review authors was involved, the quality was assessed by two independent reviewers.

The quality (in terms of the risk of bias) of all full text reviews was assessed using AMSTAR [5]. Items 1–3 and 5–8 were selected as being most important. The wording of question 7 was found to be somewhat unclear and was rephrased to “Was the **overall** scientific quality of **each** included study assessed and documented?” Thus, a yes-answer required an assessment of the overall risk of bias of each included study. The pre-specified criteria for low, moderate and high risk of bias are given in Table 1. A conservative approach was used; if a feature was not reported, it was assumed to be absent. If the answer to a particular question was unclear, it was discussed and a decision was reached in common as to whether the review should be classified as moderate or high risk of bias. Because the vast majority of published articles in the field of pediatric dentistry are identified in PubMed, it was considered acceptable to use only this database. As a general rule, the quality of individual studies in the reviews was not checked. An exception was when there was inconsistency or uncertainty about the results or conclusions of a review. In these cases, spot-test checks of individual articles were made.

If more than one systematic review on the same subject was found, only the one with the best quality and the most recent date was included [6].

**Table 1:** Criteria for assessing risk of bias.

Risk of bias	Criteria
**Low**	Predetermined research question and inclusion criteria established (AMSTAR Question 1)
	At least two independent data extractors and consensus procedure reported (AMSTAR Question 2)
	At least the database MEDLINE/Pubmed used. Search strategy reported so that it can be repeated (AMSTAR Question 3)
	A list of included and excluded studies reported (AMSTAR Question 5)
	Relevant characteristics of included studies reported (AMSTAR Question 6)
	Assessment of the overall scientific quality of each included study provided (AMSTAR Question 7)
	The scientific quality of included studies used appropriately in formulating conclusions (AMSTAR Question 8)
	The rationale for combining/not combining results reported. Methods for pooling results reported (AMSTAR Question 9)
	Likely publication bias reported. This item can be omitted if publication bias was unlikely but not reported (AMSTAR Question 10)
	Any conflict of interest reported. This item can be omitted if conflicts of interest were unlikely (AMSTAR Question 11)
Moderate	A yes-answer to questions 1, 2 and 5–8 *.
High	A no-answer to **any** of the question listed under moderate risk of bias.

Pre-specified criteria of low, moderate and high risk of bias. Modified list of questions based on AMSTAR [5].

*List of included studies is mandatory; list of excluded studies can be absent.

### Handling of data

Reviews judged as having a low or moderate risk of bias were used to summarize results and formulate existing knowledge and knowledge gaps for each domain. In accordance with the working process described by Whitlock [6], no synthesis was made of any effect size of different interventions. To get a uniform summary appraisal of the quality of evidence of the effects investigated, the various expressions used in the separate systematic reviews were transformed to GRADE terms [7]according to the following: Strong = GRADE High; Moderate = GRADE Moderate; Limited = GRADE Low; insufficient, fair, poor, low, weak, inconclusive, some evidence and other expressions of uncertainty = GRADE Very low.

## Results

The number of included reviews and the number and proportion with a low/moderate risk of bias according to the ten domains are given in Table 2. A brief summary of the objectives, main results and quality of evidence of the effects of reviews with low to moderate risk of bias is given in Table 3. It shows that the quality of evidence is high for the caries-preventive effect of daily use of fluoride toothpaste and that supervised tooth-brushing is more effective than unsupervised. Table 4 shows the current activity of published systematic reviews and original studies. The various specific outcomes related to domain are given with comments in Table 5. The main characteristics of the 38 reviews with a low or moderate risk of bias [8–45]are described in more detail in S1 Table. The 43 reviews with a high risk of bias [46–88] with the main reason for downgrading, are described in S2 Table. Due to the relatively high number of systematic reviews on prevention, this domain was subdivided into fluoride technologies, other technologies, programs/routines and safety. A summary of existing knowledge is given in Table 6 showing that existing evidence-based knowledge is limited mainly to activities for preventing caries. Knowledge gaps identified from existing reviews are summarized in Table 7. Excluded articles [89–123], with the main reason for exclusion, are listed in S3 Table.

**Table 2:** Number and distribution of included systematic reviews and number and proportion of reviews with low/moderate risk of bias according to the ten selected domains in pediatric dentistry.

Domain	Number of included reviews	Reviews with low/moderate risk of bias	Proportion with low/moderate risk of bias (%)
Behavior management problems/dental anxiety	6	3	50
Caries risk assessment and caries detection, including radiographic technologies	14	2	14
Prevention and non-operative treatment of caries in primary and young permanent teeth	43	19	44
Operative treatment of caries in primary and young permanent teeth	8	6	75
Prevention and treatment of periodontal disease	1	1	100
Management of tooth developmental and mineralization disturbances	1	1	Empty*
Prevention and treatment of oral conditions in children with chronic diseases/developmental disturbances/obesity	5	4	80
Diagnosis, prevention and treatment of dental erosion and tooth wear	0	0	No review identified
Treatment of traumatic injuries in primary and young permanent teeth	2	2	Empty
Cost-effectiveness of interventions	1	0	Empty
**Total**	**81**	**38**	**47**

* = the review did not identify any eligible studies.

**Table 3:** Brief summary of systematic reviews with low or moderate risk of bias.

Domain (number of systematic reviews)	Objectives	Main results	Quality of evidence*
Behavior management problems/dental anxiety (3)	Effect of hypnosis, pre-operative analgesics for pain relief, sedation vs general anaesthesia	Uncertain effect	Very low
Caries risk assessment (1)	Validity of multivariate models and single factors to predict caries development	Baseline caries prevalence the most accurate single predictor	Low
		Uncertain accuracy for other methods	Very low
Caries detection, including radiographic technologies (1)	Validity of methods for detecting non-cavitated caries lesions (visual, lesion activity assessment, radiography, LF, FOTI, ECM, QLF)	Acceptable diagnostic accuracy for ECM. Uncertain accuracy for other methods	Very low
Fluoride technologies for caries prevention (10)	Effects of toothpaste, varnish, mouth rinse, water fluoridation, supplements, slow release device, fluoridated food	Daily use of fluoride toothpaste effective, supervised more effective than unsupervised	High
		1500 ppm fluoride more effective than 1000 ppm	Low
		Varnish, mouth rinse, water fluoridation effective	Low
		Uncertain effect of other methods	Very low
Other technologies for caries prevention (5)	Effects of fissure sealing, HealOzone, chlorhexidine, triclosan	Fissure sealing (resin-based) effective	Moderate
		Uncertain effect of other methods	Very low
Programs/routines for caries prevention (4)	Effects of school-based programmes, recall interval, oral health promotion	Modest effect of daily tooth brushing, uncertain effect of recall interval, mass media	Very low
Non-operative treatment (2)	Effects of non-surgical methods to stop or reverse non-cavitated caries	Uncertain effect	Very low
Operative treatment (6)	Effects of restorations, excavation techniques, pulp treatment and treatment strategies for primary teeth	Uncertain effect	Very low
Prevention and treatment of periodontal disease (1)	Effects of triclosan	Uncertain effect	Very low
Management tooth developmental and mineralization disturbances (1)	Effects of restorative techniques in Amelogenesis imperfecta-affected teeth	No studies identified	Very low
Prevention and treatment of oral conditions in children with chronic diseases/developmental disturbances/obesity (4)	Relationship between chronic disease/developmental disturbances/obesity and caries	A positive relationship for asthma. No significant relationship for cleft lip/palate, chronic kidney disease or obesity	Very low
Diagnosis, prevention and treatment of dental erosion and tooth wear (0)	No systematic review identified	-	-
Treatment of traumatic injuries in primary and young permanent teeth (2)	Effects of interventions for treating external root resorption, displaced luxated front teeth	No studies identified	Very low
Cost-effectiveness of interventions (0)	No systematic review identified	-	-

*Expressions used in the systematic reviews were transformed to GRADE terms according to the following: Strong = GRADE High; Moderate = GRADE Moderate; Limited = GRADE Low; insufficient, fair, poor, low, weak, inconclusive, some evidence and other expressions of uncertainty = GRADE Very low.

Brief summary of the objectives, main results and estimated level of evidence of systematic reviews with low or moderate risk of bias. Quality of evidence of the effects according to GRADE terms [7].

**Table 4:** Distribution of systematic reviews with low or moderate risk of bias according to publication year, number of included studies and number of included studies published during the last five years.

Domain/First author/Topic	Publication year (ref no)	Studies included in the review (n)	Included studies published 2009–2014 (n)
**Behavior management problems/dental anxiety**			
Al-Harasi/Hypnosis vs sedation	2010 [8]	3	0
Ashley/Preoperative analgesics	2012 [9]	5	1
Ashley/Sedation vs general anaesthesia	2012 [10]	0	-
**Caries risk assessment and caries detection including radiographic technologies**			
Mejàre/Caries risk assessment	2014 [11]	42	16
Gomez/Caries detection	2013 [11]	42	10
**Prevention and non-operative treatment of caries in primary and young permanent teeth**			
McDonagh/Water fluoridation	2000 [13]	214	-
Ammari/Childrens´ toothpaste	2003 [14]	7	-
Twetman/Fluoride toothpaste	2003 [15]	54	-
Bonner/Slow release device	2006 [16]	1	-
Cagetti/Fluoride in food	2013 [17]	3	1
Carvalho/Varnish primary teeth	2010 [18]	8	0
Petersson/Varnish primary & permanent teeth	2004 [19]	24	-
Tubert-Jeaninn/Fluoride supplements	2011 [20]	11	0
Twetman/Fluoride mouth-rinse	2004 [21]	25	-
Yeung/Fluoride in milk	2005 [22]	2	-
Ahovuo-Saloranta/Sealants	2013 [23]	34	6
Brazelli/Heal-Ozone	2006 [24]	1	-
Hiiri/Sealant vs varnish	2010 [25]	4	0
James/Chlorhexidine	2010 [26]	12	0
Riley/Triclosan	2013 [27]	1	0
Cooper/Behavior intervention	2013 [28]	4	1
Davenport/Routine checks	2003 [29]	28	0
Kay/School programme, health promotion	1998 [30]	38	-
Riley/Recall interval	2013 [31]	1	0
Bader/Arrest non-cavitated caries	2001 [32]	22	-
Brazelli/Arrest non-cavitated caries (HealOzone)	2006 [24]	5	-
**Operative treatment of caries in primary and young permanent teeth**			
Innes/Metal crowns vs filling materials	2007 [33]	0	-
Mickenautsch/ART* vs amalgam	2010 [34]	14	0
Nadin/Pulp treatment	2003 [35]	3	-
Rasines Alcaraz/Composite vs amalgam	2014 [36]	7	0
Ricketts/Excavation techniques	2013 [37]	8	3
Yengopal/Filling materials, treatment strategies	2009 [38]	3	0
**Prevention and treatment of periodontal disease**			
Riley/ Periodontitis and triclosan	2013 [27]	1	0
**Management of tooth developmental and mineralization disturbances**			
Dashash/Amelogenesis imperfecta	2013 [39]	0	-
Alavaikko/Caries and asthma	2011 [40]	18	2
Andrade/Caries and kidney disease	2013 [41]	6	0
Hasslöf/Caries and cleft lip/palate	2007 [42]	6	-
Hayden/Caries and obesity	2013 [43]	14	5
**Treatment of traumatic injuries in primary and young permanent teeth**			
Ahangari/External root resorption	2010 [44]	0	-
Belmonte/Displaced luxated teeth	2013 [45]	0	-

* ART = atraumatic restorative technique.

**Table 5:** Main outcomes used to evaluate the effects of an intervention/diagnosis/risk assessment related to domain of systematic reviews with low or moderate risk of bias.

Domain	Main outcomes	Comments
Behavior management problems/dental anxiety	Completion of treatment, acceptance of local anaesthesia/tooth extraction, behavior, postoperative anxiety, severity or presence/absence of postoperative pain, adverse effects	Mainly qualitative outcomes
Caries risk assessment and caries detection including radiographic technologies	Predictive and diagnostic accuracy	Acceptable accuracy debatable. Patient´s benefit uncertain
Prevention and non-operative treatment of caries in primary and young permanent teeth	Caries incidence and caries lesion progression	Clinically relevant difference in effect size debatable
Operative treatment of caries in primary and young permanent teeth	Symptoms, survival of restoration/tooth, aesthetics, adverse effects	Clinically relevant difference in effect size debatable. Dichotomous success/failure may be problematic when evaluator blinding is not possible
Prevention/treatment of periodontal disease	Periodontitis (attachment loss), adverse effects	Discriminating level of attachment loss debatable
Prevention/treatment of oral conditions in children with chronic diseases/obesity/ developmental disturbances	Caries prevalence (only relationships were studied)	Clinically relevant difference debatable

Empty domains are excluded.

**Table 6:** Existing evidence-based knowledge for interventions related to pediatric dentistry.

Statement	Quality of evidence according to review authors (GRADE)
Daily use of fluoride toothpaste prevents caries; supervised tooth-brushing is more effective than unsupervised	Strong (High)
Fissure sealing with resin-based materials prevents caries on occlusal surfaces of permanent molars in individuals with high caries risk	Moderate (Moderate)
Water fluoridation reduces caries incidence	Low (Low)
Toothpaste containing 1500 ppm fluoride is more effective than 1000 ppm fluoride	Limited (Low)
Fluoride mouth rinse prevents caries if there is no additional fluoride exposure	Limited (Low)
Fluoride varnish prevents caries in permanent teeth	Limited (Low)
Baseline caries experience is the most accurate predictor of future caries	Limited (Low)

Existing evidence-based knowledge (strong, moderate or limited quality of evidence) for interventions related to pediatric dentistry.

**Table 7:** Knowledge gaps identified from the systematic reviews.

Domain	Knowledge gaps
Behavior management problems/dental anxiety	Effect of conscious sedation versus general anaesthesia
	effect of different conscious sedation techniques and dosages
	effect of pre-operative analgesics on pain relief.
Caries risk assessment and caries detection, including radiographic technologies	Validity of multivariate models and single predictors
	validity of different techniques for detecting non-cavitated caries lesions
	validity of radiographic methods for detecting enamel and dentin caries
	risk and potential harm of over- and under-detecting caries.
Prevention and non-operative treatment of caries in primary and young permanent teeth	Proper amount and level of ppm fluoride in tooth-pastes for pre-school children related to the risk of fluorosis
	effect of toothpaste introduction age, optimal brushing time and post-brushing behavior
	additional effect of fluoride mouth-rinse in high caries risk children/adolescents
	effect of fissure sealing of permanent molars in populations with low caries risk
	effect of fissure sealing of permanent molars with glass-ionomer cements
	effect of fissure sealing of permanent molars with resin-based sealants compared with glass-ionomer cements
	effect of fissure sealing compared with fluoride varnish application
	effect of fluoride varnish in primary teeth
	effect of chlorhexidine
	effects of varying other agents and methods and effect of adding fluoride to food
	effects of information, professional programs, routine dental checks and counseling
	effect of non-operative methods to arrest or reverse non-cavitated caries lesions.
Operative treatment of caries in primary and young permanent teeth	Effect of partial versus complete caries removal on signs/symptoms and restoration survival
	effects of filling materials on pain, survival and aesthetics
	effects of no treatment, non-operative or operative treatment on pain, survival and aesthetics in primary teeth
	clinical and radiographic outcome of different techniques for primary and permanent teeth with reversible pulpitis.
Prevention and treatment of periodontal disease	Effect of interventions for preventing and treating periodontal disease.
Management of tooth developmental and mineralization disturbances	Effect of interventions for managing tooth developmental and mineralization disturbances.
Prevention and treatment of oral conditions in children with chronic diseases/developmental disturbances/obesity	Effect of interventions for the management of oral conditions in children with chronic diseases/developmental disturbances/obesity and other conditions, including neuropsychiatric functional disorders and oral-motor function disturbances.
Diagnosis, prevention and treatment of dental erosion and tooth wear	Diagnostic validity and effect of interventions for preventing and treating dental erosion and tooth wear.
Treatment of traumatic injuries in primary and young permanent teeth	Effect of interventions for the management of traumatic injuries in primary and young permanent teeth.
Cost-effectiveness of interventions	Cost-effectiveness of interventions for the ten selected domains.

The main results, including existing knowledge and knowledge gaps from identified reviews with a low or moderate risk of bias, are presented below for each domain.

### Behavior management problems/dental anxiety

Three systematic reviews displayed insufficient evidence of the effect of the behavior management strategies hypnosis, use of analgesics, and sedation or general anaesthesia for the delivery of dental care [8–10]. Thus, the effects of behavior management techniques remain uncertain.

### Caries risk assessment and caries detection, including radiographic technologies

#### Caries risk assessment

One systematic review [11] concluded that comprehensive multivariate models were more accurate than single variables for predicting future caries, especially in preschool children. Few models were, however, validated. Overall, the validity of models and single risk factors, as well as the role of confounding factors (e.g. age, lifestyle, socio-economy, and socio-demography) for predicting future caries, remain uncertain.

#### Caries detection

One systematic review [12] displayed fair evidence of the accuracy of ECM (electric conductivity measurement) for detecting non-cavitated caries lesions. Poor evidence was found for all other methods, such as traditional visible inspection, bitewing radiography or other radiographic technologies and adjunct methods such as FOTI (fibre-optic transillumination), LF (laser fluorescence) and QLF (quantitative light-induced fluorescence) and lesion activity assessment (based on visual inspection).

### Prevention and non-operative treatment of caries in primary and young permanent teeth

#### Fluoride technologies for caries prevention

One systematic review concerned the caries-preventive effect of water fluoridation [13]. The quality of evidence of its effect was graded as low. There was a dose-dependent increase in dental fluorosis. Thus, the effect size of caries reduction in relation to safety remains uncertain.

Two reviews covered the preventive effect of fluoride toothpaste [14,15]. There was strong evidence for an effect of daily use of fluoride toothpaste; supervised brushing was more effective than unsupervised; evidence of a dose-dependent effect was limited. Two main uncertainties are the preventive effect in pre-school children related to the risk of fluorosis and the optimum ppm-value of fluoride in toothpastes intended for high caries risk children.

Seven reviews concerned various other fluoride technologies such as varnishes [18,19], mouth-rinses [21], slow release devices [16], tablets, drops, lozenges [20] and fluoridated food [17,22]. Whereas fluoride varnish is effective for preventing caries in permanent teeth [19], the reviews concerning primary teeth both concluded that the effect and safety of its use remain uncertain. When daily fluoride from toothpaste is used, any additional effect of fluoride mouth-rinse remains uncertain, particularly for individuals with high caries risk. The effects of all other investigated fluoride technologies also remain uncertain.

#### Other technologies for caries prevention

Five reviews covered various substances [23–27]. One addressed the effect of fissure sealants and found moderate evidence of an effect in high caries-risk children [23]. Another review compared the effect of sealants with fluoride varnish [25]. More research is needed to gain knowledge on the outcome of fissure sealants in relation to baseline caries risk, with subsequent cost-effectiveness evaluation. There are also uncertainties concerning the effect of using other than resin-based materials for sealing, pre-treatment options and any difference in effect between sealants and varnishes. Any effect of chlorhexidine, HealOzone or triclosan, also remains uncertain [24,26,27].

#### Programs/routines for caries prevention

Four reviews concerned preventive programs/routines [28–31]. Two of them reported insufficient evidence of different recall intervals [29,31]; the other two reported insufficient evidence of school-based interventions or oral health promotion programmes [28,30]. Thus, the role of programmes and routines for caries prevention, as well as the effect of recall intervals, remain uncertain.

#### Safety of using fluoride agents for caries prevention

No studies of low or moderate risk of bias regarding safety were identified. Thus, the risk of fluorosis from using fluoride toothpaste in young children (<1 or <2 years), including the amount and concentration of fluoride, remains uncertain.

#### Non-operative treatment

Two reviews concerned non-operative treatment [24,32]. One concluded that there is insufficient evidence of the efficacy of non-surgical methods (mainly fluoride supplements) to arrest or reverse non-cavitated coronal lesions [32], and the other found insufficient evidence of the effect of HealOzone for managing such lesions [24].

### Operative treatment of caries in primary and young permanent teeth

Six reviews were identified [33–38]. The effect of pre-formed metal crowns compared with filling materials in primary teeth is uncertain [33]. The most effective way of treating carious teeth also remains uncertain, i.e. the effects of stepwise, partial or no dentinal caries removal compared with complete caries removal on signs/symptoms of pulp disease and restoration failure [37]. Other uncertainties are the effect of ART compared with amalgam restorations [34], the effect of composite resin versus amalgam fillings [36] and the effect of different types of treatment for pulpally involved primary molars [35]. Furthermore, the effects of different filling materials on pain, survival and aesthetics, as well as the effects of restoration versus extraction versus no treatment in primary teeth, remain uncertain [38].

### Prevention and treatment of periodontal disease

One review on the effect of adding triclosan/copolymer to fluoride toothpaste on plaque, gingivitis, calculus and periodontitis was identified [27]. The authors concluded that adding triclosan to toothpaste had no effect on periodontitis but the statement was uncertain.

### Management of tooth developmental and mineralization disturbances

One review concluded that there is no evidence for the most effective intervention for treating teeth affected by Amelogenesis imperfecta [39]. Thus, uncertainty exists for the management of all types of mineralization disturbances as well as tooth developmental disturbances.

### Prevention and treatment of oral conditions in children with chronic diseases/developmental disturbances/obesity

Three reviews concerned dental caries prevalence/caries risk in children with asthma, chronic kidney disease or cleft lip/palate [40–42]. Although not addressing prevention, these reviews were considered important and were therefore included under this heading. All concluded that there are uncertainties concerning caries prevalence/caries risk compared with healthy children. Uncertainty also exists regarding caries risk, prevention and treatment of children with other chronic diseases, functional disabilities such as neuropsychiatric disorders and oral-motor function disturbances. One review on the relationship between obesity and dental caries concluded that the role of confounding factors remains uncertain [43].

### Diagnosis, prevention and treatment of dental erosion and tooth wear

No systematic reviews were identified.

### Treatment of traumatic injuries in primary and young permanent teeth

Two reviews were identified [44,45]. One considered the effects of interventions for treating external root resorption in permanent teeth [44] and the other the effect of treatment of displaced permanent front teeth. Both were empty reviews and any effects of these or other interventions for treating traumatic injuries therefore remain uncertain.

### Cost-effectiveness of interventions

The cost-effectiveness of different strategies for the management of dental conditions in children and adolescents remains uncertain.

## Discussion

This map report provides a systematic description of research activity in practice-relevant fields of pediatric dentistry. The effects of caries preventive strategies were relatively widely investigated and existing evidence-based knowledge was mainly restricted to this domain (Table 6). Other domains were investigated less well or not at all, resulting in a considerable number of knowledge gaps, from both existing and non-existing systematic reviews (Table 7). A possible explanation could be that existing systematic reviews were of old date. Almost two thirds of those with low or moderate risk of bias were, however, published within the latest five years (Table 4). With few exceptions the number of included studies published within the last five years was small. Some topics may be regarded as “saturated”, such as the effect of water fluoridation whereas most other topics clearly point to an urgent need for clinical research activities. In spite of several quite recently published studies, validated caries risk assessment methods are still lacking. A conceivable reason could be the complexity of the topic and the lack of consensus on methodological requirements in design, conduct, analysis and reporting. The same applies to caries detection methods where the accuracy of single or combined methods to detect non-cavitated lesions still remains a knowledge gap. The number of included studies in each systematic review shows that the major research activities during the last five years have been restricted to caries risk assessment, caries detection, fissure sealants and the relationship between obesity and caries (Table 4). The reasons for this can only be speculated on.

It follows that management of dental conditions in children and adolescents to a large extent is not evidence-based, and that at present, the best available evidence consists of own or colleagues’ experience or expert opinions. This ought to alarm stakeholders, the profession and policy-makers. It is obvious that clinical research of good quality is crucial and should be given priority so that important knowledge gaps can be eliminated. In this context it is important to note that absence of evidence of a certain intervention does not mean that there is evidence of a lack of its effect. In other words, a certain intervention may be effective even though the evidence for this is weak or lacking. The need for evidence remains, however.

The methodological quality of the systematic reviews varied and more than half of them were considered to have a high risk of bias (Table 2). The three most common shortcomings concerned questions 2, 7 and 8 in AMSTAR. A no answer to question 7 implied that each primary study was not given an overall assessment of its risk of bias. Another common reason for downgrading was that primary studies with a high risk of bias were pooled and conclusions were drawn from such results. Similarly, heterogeneous primary studies were sometimes pooled without sensitivity and subgroup analyses (question 8). The quality of evidence for a certain outcome was often not reported by the authors and if reported, the terms used varied. This made it difficult to compare the reported strength or quality of evidence of different reviews. It seems that the terms proposed by the GRADE working group (high, moderate, low and very low quality of evidence) have not yet been accepted in the literature [7]. To get a summary of the state of knowledge, reported quality of evidence of individual reviews were transformed into GRADE terms (Table 3). This was considered to be a reasonably fair way of summarizing the evidence base of individual systematic reviews.

There were six so-called empty systematic reviews, that is, there were no studies eligible for inclusion [10,33,38,39,44,45]. There is no straightforward way to assess such reviews. It has been suggested that they should be excluded [6]. We kept them, however, because they clearly point to a knowledge gap on a particular question and five of them were assessed as having a low risk of bias. One [10] was considered to have a moderate risk of bias since it may be questioned whether the inclusion criteria (RCTs) were appropriate, that is, RCTs may not be possible for ethical reasons (S1 Table).

The mapping approach is specifically designed to categorize existing literature and to identify gaps in the evidence base but it has its limitations [124]. A systematic map provides an appraisal of the methodological quality of systematic reviews but does not scrutinize the quality of the primary research included in each review. Consequently, a limitation is that individual primary studies of the systematic reviews are not scrutinized. Therefore, flaws may be overlooked, such as inconsistencies regarding the quality of individual primary studies and their qualification for contributing to synthesis and conclusions. On the other hand, as a conservative approach was taken, this limitation should not have had any major influence on the results.

The outcomes vary depending on domain (Table 5). Although well established and commonly used in dental research their robustness and clinical relevance deserves attention. The mainly qualitative outcomes in studies on behavior management problems/dental anxiety can introduce bias when interpreting the results. Thus, parents´ or children’s self-reported data may be used to decide “success” or “failure”. For example, the review by Ashley [9] points out that measures of pain depend on the baseline anxiety of the child yet none of the included studies recorded this. Regarding caries risk assessment and caries detection, the acceptable accuracy may be debatable and patient’s benefit is uncertain. The definition of a clinically relevant difference in effect size in studies on caries prevention may also be debatable. The effect size was, however, not appraised in this mapping for interventions where evidence-based knowledge exists.

It should also be noted that the external validity of the results of separate systematic reviews was not considered. Included primary research may have been undertaken in populations and settings that do not apply to today’s conditions in a particular country. An example is the statement “effective in children with high caries risk”. This might mean one thing in one country and another thing in another.

Strictly, all systematic reviews including those with high or moderate quality of evidence of the effect of a treatment displayed some gaps in knowledge. For example, there was moderate evidence that fissure sealing is effective for preventing caries. Whether that applies also to individuals with low caries risk or to other than resin-based materials still remain as knowledge gaps. So, depending on the extent of subgrouping of individuals/treatments there will probably always be gaps in knowledge. Their importance and priority for research activities must be judged accordingly.

A review of reviews aimed to assess the methodological quality of all reviews related to pediatric dentistry and oral health published by the Cochrane Oral Health Group and to assess implications for practice [125]. The authors concluded that there is strong evidence that topical fluoride treatment and sealants are effective for preventing caries in children and adolescents even though the reviews generated inconclusive findings. In contrast, the present mapping arrived at varying quality of evidence of preventive measures and identified several knowledge gaps (S1, S2 Tables and Table 7).

It is noteworthy that health-economic aspects yielded no systematic reviews of sufficient quality. One review with a high risk of bias [88] concluded that the health-economic effects of caries-preventive measures were difficult to assess due to the scarcity of original studies with sufficiently good quality and contradictory results of individual studies. A later non-systematic review on the same subject arrived at the same conclusion [112]. Overall, the cost-effectiveness needs to be addressed in future studies.

The fact that there is a severe gap in the scientific evidence on diagnosis and treatment in most fields in pediatric dentistry does not mean that there is no basis for selecting a particular method instead of another in clinical practice. For example, methods that can expose patients to large risks should be avoided. Methods involving particularly high costs should also be avoided until their cost-effectiveness has been tested properly. Furthermore, diagnosis and treatment with relevant established theoretical assumptions are preferred to methods that lack such theoretical basis. In the absence of scientific evidence for alternative methods, one should also adhere to established treatments [126]. Although important, patient-oriented aspects, such as the acceptability of an intervention, were only occasionally mentioned in the systematic reviews.

## Conclusions

There is high/moderate quality of evidence of a caries-preventive effect of daily use of fluoride toothpaste and fissure sealing with resin-based materials. For all other domains the quality of evidence of the effects of interventions was low or very low. There is an urgent need for primary clinical research of good quality in most domains in pediatric dentistry.

## Supporting Information

S1 PRISMA Checklist Reported items according to the PRISMA checklist. (DOC)

S1 Table Main characteristics of systematic reviews with low or moderate risk of bias. Main objectives, results and estimated level of evidence of systematic reviews with low or moderate risk of bias for the ten selected domains in pediatric dentistry. Presence of a knowledge gap is based on the estimated level of evidence according to authors.
(DOCX)

S2 Table Main characteristics of systematic reviews with high risk of bias. Main objectives, results and estimated level of evidence of systematic reviews with high risk of bias according to criteria listed in Table 1 for the ten selected domains in pediatric dentistry. Presence of a knowledge gap is based on the estimated level of evidence according to authors.
(DOCX)

S3 Table Excluded systematic reviews and the main reason for exclusion. (DOCX)
