# Supplementary material for: A Systematic Map of Systematic Reviews in Pediatric Dentistry—What Do We Really Know?
Source: PLoS One. 2015 Feb 23;10(2):e0117537. doi: 10.1371/journal.pone.0117537 (PMC4338212; doi:10.1371/journal.pone.0117537)
Supplement: S1 Table — Main objectives, results and estimated level of evidence of systematic reviews with low or moderate risk of bias for the ten selected domains in pediatric dentistry. Presence of a knowledge gap is based on the estimated level of evidence according to authors. (DOCX) [file pone.0117537.s002.docx]

**Table S1.** Title Main characteristics of systematic reviews with low or moderate risk of bias.

| **Behaviour management problems/dental anxiety** | | | | | | |
| --- | --- | --- | --- | --- | --- | --- |
| **First author, year, reference** | **Objectives** | **Main results according to authors** (number of studies) | | **Estimated level of evidence according to review authors** | **Knowledge gaps** | **Level of risk of bias Comments** |
| Al-Harasi, 2010 [[8](#_ENREF_8)] | Effectiveness of hypnosis for behavior management | Uncertain effect (3) | | Insufficient | Effect of hypnosis on child behavior/dental anxiety | Low |
| Ashley, 2012 [[9](#_ENREF_9)] | Effect of pre-operative analgesics for pain relief | Uncertain effect of preoperative analgesics for procedures under local anesthesia. Probable benefit prior to orthodontic separator placement (5) | | Insufficient | Effect of pre-operative analgesics for pain relief for procedures under local anesthesia | Low |
| Ashley, 2012 [[10](#_ENREF_10)] | Morbidity, effectiveness and cost-effectiveness of sedation vs general anaesthesia for delivery of dental care | No studies (0) | | Insufficient | Morbidity, effectiveness and cost-effectiveness of sedation vs general anaesthesia for delivery of dental care | Moderate |
|  |  |  |  |  |  | Inclusion criteria may be too narrow |
| **Caries risk assessment and caries detection, including radiographic technologies** | | | | | | |
| **First author, year, reference** | **Objectives** | **Main results according to authors** (number of studies) | | **Estimated level of evidence according to review authors** | **Knowledge gaps** | **Level of risk of bias Comments** |
| Mejàre, 2014 [[11](#_ENREF_11)] | Ability of multivariate models and single factors to predict caries development | Baseline caries prevalence was the most accurate single predictor in all age groups. Multivariate models and baseline caries experience performed better in pre-school children than in older children (42) | | Limited to insufficient | Validation of multivariate models and single predicting factors | Low |
|  |  |  |  |  | The role of confounding factors |  |
| Gomez, 2013 [[12](#_ENREF_12)] | Validity of methods for detecting non-cavitated caries lesions (visual, lesion activity assessment, radiography, LF, FOTI, ECM, QLF | Acceptable diagnostic accuracy for ECM (sensitivity 0. 61-0.92 and specificity 0.73-1.0) (6). Visual examination should remain standard (28) | | Fair for ECM | Accuracy of single or combination of methods to detect non-cavitated caries lesions | Low |
|  |  |  |  | Poor for other methods |  | LF= laser fluorescence, FOTI = fibre-optic transillumination, ECM=electrical caries monitoring, QLF=quantitative light-induced fluorescence |
| **Prevention and non-operative treatment of caries in primary and young permanent teeth** | | | | | | |
| **Fluoride technologies for caries prevention** | | | | | | |
| **First author, year, reference** | **Objectives** | **Main results according to authors** (number of studies) | | **Estimated level of evidence according to review authors** | **Knowledge gaps** | **Level of risk of bias Comments** |
| McDonagh, 2000 [[13](#_ENREF_13)] | Effect and safety of fluoridation of drinking water | 1)Effective for reducing caries incidence (26). Effective also with exposure to other fluoride sources (9) | | 1) Low quality of evidence | Benefits (effect size of caries reduction) related to safety (dental fluorosis) | Low |
|  | Effect when controlling for confounding factors (i.e. socio-demography and other sources of fluoride | 2)Dose-dependent increase in dental fluorosis (88) | | 2) Insufficient |  |  |
| Ammari, 2003 [[14](#_ENREF_14)] | Effect of low fluoride toothpaste ≤600 ppm vs ≥1000 ppm | Permanent teeth: | | Insufficient | Effect of ≤600 ppm compared with 1000 ppm fluoride toothpaste in pre-school children (related to water fluoridation and caries risk level) | Moderate |
|  |  | 250 ppm fluoride not effective (5) | |  |  | Excluded studies not reported |
|  |  | 500 ppm uncertain effect (2) | |  |  |  |
| Twetman, 2003 [[15](#_ENREF_15)] | Effect of fluoride toothpaste (supervised versus unsupervised tooth brushing, dose-response) in non-selected populations | Permanent teeth: | |  | Effect in pre-school children | Low |
|  |  | 1)Daily use effective (26) | | 1)Strong |  |  |
|  |  | 2)Supervised more effective than unsupervised (14) | | 2)Strong |  |  |
|  |  | 3)1500 ppm more effective than 1000 ppm (4) | | 3)Limited |  |  |
| Bonner, 2006 [[16](#_ENREF_16)] | Effect of slow-release fluoride devices | Possibly effective (1) | | Weak and unreliable | Effect of slow-release fluoride devices | Low |
| Cagetti, 2013 [[17](#_ENREF_17)] | Effects of fluoridated food (milk, sugar, salt) | Effectiveness uncertain for milk (2) | | Low for milk | Effect of fluoridated food (milk, sugar, salt) | Moderate |
|  |  | Salt (0) | | None for sugar and salt |  | Unclear search strategy |
|  |  | Sugar (1) | |  |  |  |
| Carvalho, 2010 [[18](#_ENREF_18)] | Effect of fluoride varnish for preventing caries in primary teeth | Possibly effective for reducing caries incidence (8) | | No conclusive evidence | Effect in primary teeth. Safety | Low |
| Petersson, 2004 [[19](#_ENREF_19)] | Effectiveness and safety of fluoride varnish in permanent and primary teeth. Frequency of application | Effective in permanent teeth (21), | | Limited for permanent and | Effect in primary teeth. | Low |
|  |  | Uncertain for primary teeth (3) | | Inconclusive for primary teeth | Application frequency. |  |
|  |  |  | | Insufficient | Safety |  |
| Tubert-Jeaninn,2011 [[20](#_ENREF_20)] | Effect of fluoride supplements (tablets, drops, lozenges or chewing gum) compared with no or with topical fluorides | Compared with no fluoride supplement: Reduction in caries increment in permanent teeth (9), unclear for primary teeth (4) | | Weak | Effect of fluoride supplements with or without use of topical fluorides (toothpaste, mouth-rinse). | Low |
|  |  | Compared with topical fluorides (5): no effect | |  | Adverse effects (fluorosis) |  |
| Twetman 2004 [[21](#_ENREF_21)] | Effect of fluoride mouth-rinses with or without other fluoride sources (piped water, toothpaste) | 1)Effective without additional fluoride exposure (8) | | 1)Limited | Additional effect of fluoride mouth-rinse in children and adolescents with high caries risk | Low |
|  |  | 2)Additional effect from other fluoride sources questionable (15) | | 2)Weak (inconclusive) |  |  |
| Yeung, 2005 [[22](#_ENREF_22)] | Effectiveness of fluoridated milk in primary and permanent teeth | May be effective in young permanent teeth (2) | | Insufficient | Effect of fluoridated milk | Low |
| **Safety of using fluoride agents for caries prevention** | | | | | | |
| No systematic reviews identified | | | | | | |
| **Other technologies for caries prevention** | | | | | | |
| **First author, year, reference** | **Objectives** | **Main results according to authors** (number of studies) | | **Estimated level of evidence according to review authors** | **Knowledge gaps** | **Level of risk of bias Comments** |
| Ahovuo-Saloranta, 2013 [[23](#_ENREF_23)] | To compare the effects of different types of fissure sealants: | 1)Sealing permanent molars’ occlusal surfaces is effective in high-risk individuals up to 48 months (4). | | 1)Moderate | Effectiveness of sealants related to baseline caries prevalence. | Low |
|  | Effectiveness related to baseline caries prevalence | 2)Uncertain benefit of sealing under other conditions (3). | | 2)Very low | Effects of glass ionomer cement materials compared with resin-based sealants |  |
|  | Effectiveness of different sealant materials | 3)Uncertain effect of other than resin-based materials (8) | | 3)Very low |  |  |
|  | Adverse effects | 4)No adverse effects (2) | | 4)Not stated |  |  |
| Brazzelli, 2006 [[24](#_ENREF_24)] | Effectiveness and cost-effectiveness of Heal ozone | No significant benefit on pit and fissure caries (1) | | Insufficient | Effectiveness and cost-effectiveness of ozone | Low |
| Hiiri, 2010 [[25](#_ENREF_25)] | Effectiveness of pit and fissure sealants compared with fluoride varnishes | Pit and fissure sealants more effective than fluoride varnishes (4) | | Some evidence | Effectiveness of pit and fissure sealants compared with fluoride varnishes | Low |
| James, 2010 [[26](#_ENREF_26)] | Effect of chlorhexidine varnish | Permanent teeth: | | Inconclusive | Effect of chlorhexidine varnish | Moderate |
|  | Effect compared with fluoride varnish | No effect vs placebo/ no treatment (8), effective (2) | |  | Effect compared with fluoride varnish | Unclear search strategy |
|  |  | Primary teeth: inconclusive results (2) | |  |  |  |
| Riley, 2013 [[27](#_ENREF_27)] | Effect of triclosan/copolymer- containing fluoride toothpastes, compared with fluoride toothpaste on caries | No effect on caries in children of triclosan/copolymer- containing fluoride toothpastes (1) | | Insufficient | Effect of triclosan/copolymer- containing fluoride toothpastes on caries incidence in children | Low |
| **Programs/routines for caries prevention** | | | | | | |
| **First author, year, reference** | **Objectives** | **Main results according to authors** (number of studies) | | **Estimated level of evidence according to review authors** | **Knowledge gaps** | **Level of risk of bias Comments** |
| Cooper, 2013 [[28](#_ENREF_28)] | Efficacy of school-based behavioral interventions | Effect on caries development (1) | | Insufficient | Effect of school-based interventions for changing behavior and reducing caries development | Low |
| Davenport, 2003 [[29](#_ENREF_29)] | Effects of routine dental checks of different recall intervals | Relative effectiveness of different frequencies of dental checks not possible to assess (28) | | No high quality evidence | Effects of routine dental checks of different recall intervals | Low |
| Kay, 1998 [[30](#_ENREF_30)] | Effects of oral health promotion | Modest effect of tooth-brushing programs at school (7) | | Insufficient | Effects of oral health promotion, both chair-side and from mass media | Low |
|  |  | Uncertain effect on plaque of tooth-brushing instruction ([23]) | |  |  |  |
|  |  | Unclear effect of mass media oral health promotion (8) | |  |  |  |
| Riley, 2013 [[31](#_ENREF_31)] | Effect on oral health and the economic impact of different recall intervals (for example six versus 12 months) | Not enough known to determine the most effective recall interval between dental check-ups (1) | | Insufficient | Potential beneficial and harmful effects of varying recall intervals between dental check-ups | Low |
| **Non-operative treatment** | | | | | | |
| **First author, year, reference** | **Objectives** | **Main results according to authors** (number of studies) | | **Estimated level of evidence according to review authors** | **Knowledge gaps** | **Level of risk of bias Comments** |
| Bader, 2001[[32](#_ENREF_32)] | Efficacy of non-surgical methods to stop or revers non-cavitated coronal caries | Not enough known to determine the efficacy of the methods (7) | | Insufficient | Efficacy of non-surgical methods (mainly fluoride supplements) to stop or revers non-cavitated coronal caries | Moderate Excluded studies not reported |
| Brazzelli, 2006 [[24](#_ENREF_24)] | The effectiveness and cost-effectiveness of HealOzone for managing non-cavitated pit and fissure caries and root caries | Not possible to judge effectiveness.Costs higher for sealants but lower for non-cavitated root caries (5 + 5 abstracts) | | Insufficient | Effectiveness and cost-effectiveness of ozone for managing non-cavitated pit and fissure caries | Low |
| **Operative treatment of caries in primary and young permanent teeth** | | | | | | |
| **First author, year, reference** | **Objectives** | **Main results according to authors** (number of studies) | | **Estimated level of evidence according to review authors** | **Knowledge gaps** | **Level of risk of bias Comments** |
| Innes, 2007 [[33](#_ENREF_33)] | Effect on clinical outcomes of preformed metal crowns compared with filling materials in primary teeth | No studies identified (0) | | Insufficient | Effect on clinical outcomes of preformed metal crowns compared with filling materials in primary teeth | Low |
| Mickenautsch, 2010 [[34](#_ENREF_34)] | Longevity of atraumatic restorative treatment (ART) compared with equivalent amalgam restorations | Equal longevity of ART in primary teeth compared with equivalent amalgam restorations. Equal or greater longevity in permanent teeth (14) | | Insufficient | Longevity of Class I, II and V restorations using the ART approach compared with equivalent amalgam restorations | Low |
| Nadin, 2003 [[35](#_ENREF_35)] | Effects of various pulp treatment techniques in retaining primary molars with decay involving the pulp for at least 12 months | Data unavailable on long-term effects of treatments (3) | | Insufficient | Effects of different types of treatment for pulpally involved primary molars | Low |
| Rasines Alcaraz, 2014 [[36](#_ENREF_36)] | Effect of direct composite resin fillings versus amalgam fillings in posterior permanent teeth primarily on restoration failure | Composite resin fillings almost twice as likely to fail or have secondary caries compared with amalgam fillings (10) | | Low | Adverse effects of amalgam on patients .Long-term effect of the latest improved composite materials, techniques and instruments for placing them | Low |
| Ricketts, 2013 [[37](#_ENREF_37)] | Effects of stepwise, partial or no dentinal caries removal vs complete caries removal on signs/symptoms of pulp disease and restoration failure | Partial or stepwise caries removal reduces the incidence of pulp exposure (8) | | Insufficient | The most effective intervention for treating carious teeth: | Low |
|  |  |  |  |  | No, partial or complete caries removal |  |
|  |  |  |  |  | Restoration failure related to these alternatives |  |
| Yengopal, 2009 [[38](#_ENREF_38)] | Effects in primary teeth of: |  | | Insufficient | Effects in primary teeth of: | Low |
|  | 1) Pain, survival and aesthetics of filling material | 1)No differences between filling materials (3) | |  | Type of filling material on pain, survival and aesthetics |  |
|  | 2) Restoration vs extraction vs no treatment | 2) No studies identified (0) | |  | Restoration vs extraction vs no treatment |  |
| **Prevention and treatment of periodontal disease** | | | | | | |
| **First author, year, reference** | **Objectives** | **Main results according to authors** (number of studies) | | **Estimated level of evidence according to review authors** | **Knowledge gaps** | **Level of risk of bias Comments** |
| Riley, 2013 [[27](#_ENREF_27)] | Effect of triclosan/copolymer- containing fluoride toothpastes, compared with fluoride toothpaste on plaque, calculus, gingivitis and periodontitis | No effect on periodontitis (1) | | Insufficient | Effect of triclosan/copolymer containing fluoride toothpastes for preventing periodontitis | Low |
| **Management of tooth developmental and mineralization disturbances** | | | | | | |
| **First author, year, reference** | **Objectives** | **Main results according to authors** (number of studies) | | **Estimated level of evidence according to review authors** | **Knowledge gaps** | **Level of risk of bias Comments** |
| Dashash, 2013 [[39](#_ENREF_39)] | Effects on success rate of restorative techniques in Amelogenesis imperfecta-affected teeth | No RCT identified (0) | | None | The most effective interventions for Amelogenesis imperfecta-affected teeth | Low |
| **Prevention and treatment of oral conditions in children with chronic diseases/developmental disturbances/obesity** | | | | | | |
| **First author, year, reference** | **Objectives** | **Main results according to authors** (number of studies) | | **Estimated level of evidence according to review authors** | **Knowledge gaps** | **Level of risk of bias Comments** |
| Alavaikko, 2011 [[40](#_ENREF_40)] | Relationship between asthma and dental caries | Asthma doubles the risk of caries in both primary and permanent teeth (18) | | Inconclusive | Level of caries risk in primary and permanent teeth in children with asthma | Low |
| Andrade, 2013 [[41](#_ENREF_41)] | Dental caries prevalence in children with chronic kidney disease compared with healthy children | Weak evidence that children with chronic kidney disease have lower caries prevalence than healthy children (6) | | Weak | Caries prevalence in children with chronic kidney disease compared with healthy children | Moderate Unclear search strategy |
| Hasslöf, 2007 [[42](#_ENREF_42)] | Dental caries prevalence in children with cleft lip and or palate compared with non-cleft controls | Inconsistent findings: | | Insufficient | Caries prevalence in children with cleft lip and or palate compared with children without cleft lip and or palate | Moderate |
|  |  | No signifcant difference (4); significant difference (2) | |  |  | Only one database |
| Hayden, 2013 [[43](#_ENREF_43)] | The relationship between obesity and dental caries | There is a non-significant positive relationship between obesity and dental caries in the permanent dentition (14) | | Not stated (Insufficient) | The role of confounding factors (i.e. age, lifestyle, socioeconomy and demography) | Low |
| **Diagnosis, prevention and treatment of dental erosion and tooth wear** | | | | | | |
| No systematic reviews identified | | | | | | |
| **Treatment of traumatic injuries in primary and young permanent teeth** | | | | | | |
| **First author, year, reference** | **Objectives** | **Main results according to authors** (number of studies) | **Estimated level of evidence according to review authors** | | **Knowledge gaps** | **Level of risk of bias Comments** |
| Ahangari, 2010 [[44](#_ENREF_44)] | Effectiveness of interventions for the management of external root resorption in permanent teeth | No studies matching inclusion criteria identified (0) | Insufficient | | Effect of interventions for the management of external root resorption in permanent teeth | Low |
| Belmonte, 2013 [[45](#_ENREF_45)] | Effects of a range of interventions for treating displaced luxated permanent front teeth | No randomized or quasi-randomized controlled trials identified (0) | Insufficient | | Effect of treatment of displaced luxated permanent front teeth | Low |
| **Cost-effectiveness of interventions** | | | | | | |
| No systematic reviews identified | | | | | | |

Table S1 legend. Main objectives, results and estimated level of evidence of systematic reviews with low or moderate risk of bias for the ten selected domains in pediatric dentistry. Presence of a knowledge gap is based on the estimated level of evidence according to authors.
