# Supplementary material for: A Systematic Map of Systematic Reviews in Pediatric Dentistry—What Do We Really Know?
Source: PLoS One. 2015 Feb 23;10(2):e0117537. doi: 10.1371/journal.pone.0117537 (PMC4338212; doi:10.1371/journal.pone.0117537)
Supplement: S2 Table — Main objectives, results and estimated level of evidence of systematic reviews with high risk of bias according to criteria listed in Table 1 for the ten selected domains in pediatric dentistry. Presence of a knowledge gap is based on the estimated level of evidence according to authors. (DOCX) [file pone.0117537.s003.docx]

**Table S2.** **Title** Main characteristics of systematic reviews with high risk of bias.

| **Behaviour management problems/dental anxiety** | | | | | |
| --- | --- | --- | --- | --- | --- |
| **First author, year, reference** | **Objectives** | **Main results according to authors** (number of studies) | **Estimated level of evidence according to review authors** | **Knowledge gaps** | **A no answer to questions # 1,2, 5-8 Comments** |
| Lourenco-Matharu, 2012 [[46](#_ENREF_46)] | Efficacy of conscious sedation agents and dosages for behavior management | Uncertain effect of Midazolam (5) and | Weak for midazolam | Effect of midazolam, nitrous oxide and other agents on child behavior/dental anxiety | # 7, 8 |
|  |  | nitrous oxide oxygen sedation (2) | Very weak for nitrous oxide oxygen sedation |  |  |
| Matharu, 2006 [[47](#_ENREF_47)] | Efficacy of conscious sedation techniques for behavior management | Uncertain effect (61) | Insufficient | Effect of conscious sedation techniques on child behavior/dental anxiety | # 7, 8 |
| Zhou, 2011 [[48](#_ENREF_48)] | Effect of dental staff behavior on child dental patient anxiety and behavior | Children’s’ anxiety and/or behavior may be influenced by certain staff behaviors (11) | Insufficient | Effects of dental staff behavior on child´s anxiety and behavior during and after treatment | # 7, 8 |
| **Caries risk assessment and caries detection, including radiographic technologies** | | | | | |
| **First author, year, reference** | **Objectives** | **Main results according to authors** (number of studies) | **Estimated level of evidence according to review authors** | **Knowledge gaps** | **A no answer to questions # 1,2, 5-8 Comments** |
| Bloemendal, 2004 [[49](#_ENREF_49)] | Additional value of radiographic compared with clinical examination to assess caries prevalence | Radiographic examination has an unknown additional value to assess caries prevalence (7) | Not stated | Validity and additional value of radiographic examination to assess caries prevalence | # 2,7,8 |
| Burt, 2001 [[50](#_ENREF_50)] | Does low birth weight increase the risk of caries? | No relationship reported (4) | Weak for primary teeth | Relationship between low birth weight and caries in primary and permanent teeth | # 5,6,8 |
|  |  |  | No evidence for permanent teeth |  |  |
| Harris, 2004 [[51](#_ENREF_51)] | Risk factors for dental caries in young children | 106 risk factors with significant relationship to caries (77) | Insufficient | Longitudinal studies on risk factors for dental caries | # 2,5, 8 |
| Hooley, 2012 [[52](#_ENREF_52)] | The relationship between body mass index and dental caries | Positive associations with both high and low BMI (47) | Limited | The role of body mass index on caries risk | #2 |
| Ismail, 1999 [[53](#_ENREF_53)] | Diagnostic criteria of early childhood caries | No consensus in case definitions and diagnostic criteria (94) | Insufficient | Validated diagnostic tools to define and study ECC etiology and epidemiology | # 2,7,8 |
| Leong, 2012 [[54](#_ENREF_54)] | Determinants of ECC during first year of life (maternal factors, colonization mediated by oral health behaviors, practise and feeding habits | There is a non-significant positive relationship between obesity and dental caries in the permanent dentition (14) | Limited to insufficient | The role of bacterial colonization in the caries process | #8 |
| Parisotto, 2010 [[55](#_ENREF_55)] | ECC and mutans streptococci | Mutans streptococci levels are a strong risk indicator for ECC (16) | Limited | ECC and mutans streptococci colonization. Prospective studies of high quality are lacking | #2 |
| Reisine, 2001 [[56](#_ENREF_56)] | Socioeconomic status and behavior determinants as risk factors for dental caries | An inverse relationship between SES and caries in children under 12 years (272) | Fairly strong | Socioeconomic status and behavior determinants as risk factors for dental caries in adolescents | # 2,8 |
| Thenisch, 2006 [[57](#_ENREF_57)] | Association between Mutans streptococci and caries incidence i preschool children | Presence of MS in plaque or saliva associated with considerable caries risk (7) | Limited to Insufficient for the strength of association | The role of confounding factors | # 7,8 |
| Tellez, 2012 [[58](#_ENREF_58)] | Predictive ability of four caries risk assessment systems | Only Cariogram validated in prospective trials, albeit limited reliability (14) | Limited | Valid and reliable methods for caries risk assessment | # 8 |
| Twetman, 2013 [[59](#_ENREF_59)] | Accuracy of adjunct methods ( FOTI, Di FOTI, QLF, LF and ECM) to detect and quantify enamel and dentin caries lesions | Limited evidence for accuracy of LF (12) and ECM (4). | Limited to insufficient | Accuracy of adjunct methods to detect and quantify enamel and dentin caries lesions. | # 8 |
|  |  | Insufficient evidence for FOTI, DiFOTI (4) and QLF (0). |  | Cost-effectiveness of the methods. |  |
|  |  | Adjunct methods should not be used alone |  |  |  |
| Valaitis, 2000 [[60](#_ENREF_60)] | Association between breastfeeding and early childhood caries | An association may exist between breastfeeding over one year at night, but results are inclusive (28) | Equivocal | Relevance of weaning to prevent early childhood caries | # 5,6 |
| **Prevention and non-operative treatment of caries in primary and young permanent teeth** | | | | | |
| **Fluoride technologies for caries prevention** | | | | | |
| **First author, year, reference** | **Objectives** | **Main results according to authors** (number of studies) | **Estimated level of evidence according to review authors** | **Knowledge gaps** | **A no answer to questions #1,2, 5-8 Comments** |
| Azarpazhooh, 2008 [[61](#_ENREF_61)] | Effect of fluoride varnish in high-risk children, particularly young children | Effective for at least two applications per year (7). | Clear evidence | Effect in high risk children | # 8 |
|  |  |  |  |  | Unclear # 1,5 |
| Espelid, 2009 [[62](#_ENREF_62)] | Effects of fluoride in milk, salt, tablets and lozenges | Fluoride tablets/drops may be effective (7). | Limited for fluoride tablets Insufficient for milk | Effects of fluoride in milk, salt and tablets | # 2 |
| Ismail, 2008 [[63](#_ENREF_63)] | 1. Effect of fluoride supplements (tables, drops, lozenges) | Effective in primary teeth (12). | 1.Weak | Effects of tablets, drops, lozenges | # 7,8 Unclear #2 |
|  |  |  |  |  |  |
|  | 2.Risk of fluorosis | Increased risk of fluorosis, especially during 1^st^ three years (5) | 2.Level not stated | Safety |  |
| Marinho, 2003 [[64](#_ENREF_64)] | Effect of fluoride toothpaste | 1. Permanent teeth: Unsupervised: Effective if at least once per day (56). Twice per day (7). | 1. Strong | Effect in primary teeth | # 2 |
|  | Effect of super vised tooth brushing | 2. Supervised (18) increased the benefit. | 2. Evidence level not reported | Adverse effects |  |
|  |  | 3. Primary teeth: Effective (1) | 3. Insufficient |  |  |
| Marinho, 2003 [[65](#_ENREF_65)] | Effect of adding topical fluorides (mouth-rinses, gels, or varnishes) compared with toothpaste used alone | Relatively small caries reduction (PF=10 %) by adding topical fluorides to fluoride toothpaste use (12) | Evidence level not reported | Effect for children at higher caries risk | # 2 |
|  |  |  | “Cautious interpretation of data” | Effect in pre-school children |  |
| Marinho, 2003 [[66](#_ENREF_66)] | Effect of topical fluorides (toothpastes, mouth-rinses, gels or varnishes) and potentially modifying factors | Firmly established benefit of topical fluorides (28). | Evidence level not reported | Effect in pre-school children Adverse effects | # 2,8,9 |
| Marinho, 2003 [[67](#_ENREF_67)] | Effectiveness, safety and acceptability of fluoride mouth-rinses and effect of modifying factors | Regular and supervised fluoride rinse is associated with reduction in caries increment (34). | Strong | Safety and acceptability of supervised mouth-rinsing | # 2 |
|  |  | Less effective in individuals with low caries increment. |  | Effects with regular use of fluoride toothpaste |  |
| Marinho, 2003 [[68](#_ENREF_68)] | Effectiveness of one topical fluoride intervention vs another (toothpaste, mouth-rinse, varnish and gel) | 1) Topical fluorides (mouth-rinses or gels no more effective than fluoride toothpaste (10). | 1) Level not reported | Adverse effects | # 2 |
|  |  | 2) No strong evidence that fluoride varnish is more effective than other types of topical fluoride (5). | 2) Inconclusive |  |  |
| Marinho, 2002 [[69](#_ENREF_69)] | Effect of fluoride gels related to initial caries level, background exposure to fluoride, mode and frequency of use | Effective in permanent teeth (23) | Clear evidence | Effect in deciduous teeth. Effect when fluoride toothpaste is also used | # 2 |
|  |  | No relationship between effect and studied covariates. | Uncertain | Safety and acceptability |  |
| Marinho, 2013 [[70](#_ENREF_70)] | Effectiveness and safety of fluoride varnish in primary and permanent teeth | Effective in both primary and permanent teeth when applied 2-4 times/year (22). | Moderate | Safety and acceptability | # 7,8 |
| Santos, 2013 [[71](#_ENREF_71)] | Effects of low (<600 ppm) vs standard (1000-1500 ppm) toothpaste on caries incidence and dental fluorosis | Standard toothpastes more effective than low ppm toothpastes (3). | No evidence to support the use of low ppm fluoride toothpaste | Safety of using standard toothpaste in pre-school children | #8 Unclear # 7 |
|  |  | Low ppm toothpaste did not significantly reduce the risk of dental fluorosis (2). |  |  |  |
| Santos, 2013 [[72](#_ENREF_72)] | Effect of fluoride toothpaste in pre-school children (primary teeth) | Standard toothpastes (1000-1500 ppm) effective (8). | Level not stated Evidence that standard toothpaste (1000-1500 ppm) is effective in primary teeth | Effect of <600 ppm compared with 1000 ppm fluoride toothpaste in pre-school children | # 7,8 |
|  |  | <600 ppm toothpastes effective only at surface level (2) |  |  |  |
| Walsh, 2010 [[73](#_ENREF_73)] | Effect of fluoride toothpaste with different concentrations and modifying effects (baseline caries, supervised brushing) | Some evidence of dose-response relationship. |  | Effect of different concentrations of fluoride in toothpastes for pre-school children. | # 7,8 |
|  |  | 1) 250 ppm less effective than 1000 ppm (2). | 1) Weak | Safety in pre-school children |  |
|  |  | 2) 500 ppm less effective than 1100 ppm (2). | 2) Very weak |  |  |
|  |  | 3) 1000 ppm less effective than higher ppm values up to 1450 ppm (6). | 3) Strong |  |  |
| Wright, 2014 [[74](#_ENREF_74)] | Effect of fluoride toothpaste in children younger than 6 years on: |  | 1.and 2: Limited | Safety of using fluoride toothpaste in pre-school children | # 7, 8 |
|  | 1. Caries | 1. Fluoride toothpaste is effective in caries control (8) |  |  |  |
|  | 2. Dental fluorosis | 2. Ingesting pea-sized amounts or more can lead to mild fluorosis (5). |  |  |  |
| **Safety of using fluoride agents for caries prevention** | | | | | |
| **First author, year, reference** | **Objectives** | **Main results according to authors** (number of studies) | **Estimated level of evidence according to review authors** | **Knowledge gaps** | **A no answer to questions # 1, 2, 5-8 Comments** |
| Wong, 2010 [[75](#_ENREF_75)] | Risk of mild fluorosis from fluoride toothpaste with different concentrations and amount of paste applied | Higher risk when starting before 12 months of age (4) | Weak, unreliable | Risk of mild fluorosis from use of fluoride toothpaste at ages 1 to 2. | # 8 |
|  |  | Equivocal when starting between 12 and 24 months of age (5). |  |  | (Some conclusions based on cross-sectional and retrospective studies) |
|  |  | No association between fluorosis and frequency of brushing (4) or amount of fluoride (3). |  |  |  |
| **Other technologies for caries prevention** | | | | | |
| **First author, year, reference** | **Objectives** | **Main results according to authors** (number of studies) | **Estimated level of evidence according to review authors** | **Knowledge gaps** | **A no answer to questions # 1,2, 5-8 Comments** |
| Hujoel, 2013 [[76](#_ENREF_76)] | Effect of vitamin D | Vitamin D may reduce caries incidence (24) | Insufficient | Effect of vitamin D on caries incidence | # 1,2,8 |
| Hujoel, 2006 [[77](#_ENREF_77)] | Effect of self-performed dental flossing on proximal caries | No effect (2) | Insufficient | Effect of self-performed flossing related to fluoride use | # 2,7,8 |
|  | Effect of professional dental flossing on proximal caries | Professional flossing at schooldays is effective in children with low fluoride exposure (5) | Insufficient | Effect of professional flossing related to fluoride use |  |
| Moynihan, 2014 [[78](#_ENREF_78)] | 1. The effect on dental caries of increasing or decreasing the amount of sugars intake. | 1. Positive association between sugars and caries. | Moderate | The effect on caries of reducing sugars through dietary interventions and health education | #7,8 |
|  | 2. Does the evidence support a threshold for sugars intake? | 2. There may be benefit in limiting sugars to < 10% energy (55) |  |  |  |
| Twetman, 2012 [[79](#_ENREF_79)] | Effect of probiotics | Probiotic bacteria may interfere with oral biofilm (19) | Not reported | Effect of probiotics | # 2,7,8 |
| Zhang, 2006 [[80](#_ENREF_80)] | Effect of chlorhexidine varnish and application frequency in permanent teeth | A moderate caries-inhibiting effect when applied every 3-4 months (14) | Insufficient for lasting effect | Effect of chlorhexidine varnish and application frequency in permanent teeth | # 2,8 |
| **Programs/routines for caries prevention** | | | | | |
| **First author, year, reference** | **Objectives** | **Main results according to authors** (number of studies) | **Estimated level of evidence according to review authors** | **Knowledge gaps** | **A no answer to questions # 1,2, 5-8 Comments** |
| Ammari, 2007 [[81](#_ENREF_81)] | Effects of professional programs for 0-5 yr-olds | No evidence of dental health education (1) | Not clearly stated | Best professional program for preventing early childhood caries | # 5,7,8 |
|  |  | Fluoride-based intervention effective (5) |  |  |  |
| Bader, 2004 [[82](#_ENREF_82)] | Effects of physicians´ intervention (e.g. fluoride varnish application, screening and risk assessment, referral and counseling) in pre-school children | Fluoride supplements (6) and varnish (6) effective | Fair for fluoride supplements and varnish | Effects of physicians´ intervention in pre-school children | # 2,7,8 |
|  |  | Screening, risk assessment, referral, counselling uncertain effect (12) | No for screening, referral and counselling |  |  |
| Bhaskar, 2014 [[83](#_ENREF_83)] | Effectiveness of early preventive dental visits in improving children’s oral health outcomes. | Mixed or no support for the effectiveness of early preventive dental visits (4) | Weak | Effectiveness of early preventive dental visits | # 7, 8 |
| **Non-operative treatment** | | | | | |
| **First author, year, reference** | **Objectives** | **Main results according to authors** (number of studies) | **Estimated level of evidence according to review authors** | **Knowledge gaps** | **A no answer to questions # 1,2, 5-8 Comments** |
| Tellez, 2012 [[84](#_ENREF_84)] | Efficacy of non-surgical methods to arrest or reverse progression of non-cavitated caries lesions (Fluoride-varnish, gel, toothpaste, CHX, xylitol, CCP-ACP, sealant, resin infiltration) | Fluorides effective, Potential for sealants and resin infiltration to slow down or reverse lesion progression(29) | Insufficient, except for fluoride and sealants | Effects of CCP-ACP gum, CHX, xylitol, sealant or resin infiltration to arrest or reverse progression of non-cavitated caries lesions | # 8 |
| **Operative treatment of caries in primary and young permanent teeth** | | | | | |
| **First author, year, reference** | **Objectives** | **Main results according to authors** (number of studies) | **Estimated level of evidence according to review authors** | **Knowledge gaps** | **A no answer to questions # 1,2, 5-8 Comments** |
| Ferreira, 2012 [[85](#_ENREF_85)] | Effect of sealing or limited removal of carious tissue for arresting lesions in primary teeth | Partial or non-mechanical removal of carious tissue can arrest caries in primary teeth (3) | Limited | Effect of partial removal of carious tissue to arrest caries lesions in primary teeth | # 2,7,8 |
| Simarcas-Pallares, 2010 [[86](#_ENREF_86)] | Clinical and radiographic effects of pulpotomy using formocresol or mineral trioxide (MTA) aggregate in primary teeth | Both successful but authors suggest MTA due to FC cytotoxicity (6) | Insufficient | Clinical and radiographic outcome of different techniques for primary molars with reversible pulpitis | # 6,7,8 |
|  |  |  |  |  | #2 unclear |
| **Prevention and treatment of periodontal disease** | | | | | |
| No systematic reviews identified | | | | | |
| **Management of tooth developmental and mineralization disturbances** | | | | | |
| No systematic reviews identified | | | | | |
| **Prevention and treatment of oral conditions in children with chronic diseases/developmental disturbances/obesity** | | | | | |
| **First author, year, reference** | **Objectives** | **Main results according to authors** (number of studies) | **Estimated level of evidence according to review authors** | **Knowledge gaps** | **A no answer to questions # 1,2, 5-8 Comments** |
| Chi, 2013 [[87](#_ENREF_87)] | Dental caries prevalence in children with cystic fibrosis vs healthy children | Uncertain whether children with cystic fibrosis have an increased caries risk compared to healthy children (15) | Insufficient | Caries prevalence in children with cystic fibrosis compared to healthy children. | # 2 |
| **Diagnosis, prevention and treatment of dental erosion and tooth wear** | | | | | |
| No systematic reviews identified | | | | | |
| **Treatment of traumatic injuries in primary and young permanent teeth** | | | | | |
| No systematic reviews identified | | | | | |
| **Cost-effectiveness of interventions** | | | | | |
| **First author, year, reference** | **Objectives** | **Main results according to authors** (number of studies) | **Estimated level of evidence according to review authors** | **Knowledge gaps** | **A no answer to questions # 1,2, 5-8 Comments** |
| Källestål, 2003 [[88](#_ENREF_88)] | Economic evaluation of caries preventive methods | No support for the economic value of caries prevention (17) | Insufficient | Health economic effects of caries-preventive methods/programs | # 2 |

Table S2 legend. Main objectives, results and estimated level of evidence of systematic reviews with high risk of bias according to criteria listed in Table 1 for the ten selected domains in pediatric dentistry. Presence of a knowledge gap is based on the estimated level of evidence according to authors.
