# Supplementary material for: A Systematic Map of Systematic Reviews in Pediatric Dentistry—What Do We Really Know?
Source: PLoS One. 2015 Feb 23;10(2):e0117537. doi: 10.1371/journal.pone.0117537 (PMC4338212; doi:10.1371/journal.pone.0117537)
Supplement: S3 Table — (DOCX) [file pone.0117537.s004.docx]

**Table S3.** Excluded systematic reviews and the main reason for exclusion.

| **First author, year, reference** | **Main reason for exclusion** |
| --- | --- |
| Aartman, 1998 [[89](#_ENREF_89)] | Not a systematic review |
| Ahovuo-Saloranta, 2008 [[90](#_ENREF_90)] | Updated 2013 |
| Ahovuo-Saloranta, 2004 [[91](#_ENREF_91)] | Updated 2013 |
| Aleksejuiene, 2013 [[92](#_ENREF_92)] | Not a systematic review |
| Alves, 2014 [[93](#_ENREF_93)] | Not relevant |
| Azarpazhooh, 2008 [[94](#_ENREF_94)] | Duplicate publication |
| Azarpazhooh, 2009 [[95](#_ENREF_95)] | Duplicate publication |
| Azarpazhooh, 2009 [[96](#_ENREF_96)] | Not a systematic review |
| Bader, 2006 [[97](#_ENREF_97)] | Not a systematic review |
| Beirne, 2007 [[98](#_ENREF_98)] | Update available |
| Brocklehurst, 2013 [[99](#_ENREF_99)] | Not relevant |
| Chou, 2013 [[100](#_ENREF_100)] | Update of guidelines |
| de Castilho, 2013 [[102](#_ENREF_102)] | Not a systematic review |
| Delpier, 2013 [[101](#_ENREF_101)] | Not a systematic review |
| Font-Ribera 2013 [[104](#_ENREF_104)] | Not relevant |
| Frencken, 2012 [[103](#_ENREF_103)] | Not a systematic review |
| Gawade, 2014 [[105](#_ENREF_105)] | Not relevant |
| Haugejorden, 1996 [[106](#_ENREF_106)] | Not a systematic review |
| Ijaz, 2010 [[107](#_ENREF_107)] | Review of reviews |
| Kantovitz, 2006 [[108](#_ENREF_108)] | Update available |
| Khan, 2014 [[109](#_ENREF_109)] | Not relevant |
| Llordra, 1993 [[110](#_ENREF_110)] | Update available |
| Marinho, 2002 [[70](#_ENREF_70)] | Updated 2013 |
| Marinho, 2009 [[114](#_ENREF_114)] | Review of reviews |
| Marino, 2013 [[112](#_ENREF_112)] | Not a systematic review |
| Matharu, 2005 [[113](#_ENREF_113)] | Updated 2006 |
| Mejàre, 2003 [[115](#_ENREF_115)] | Update available |
| Ribeiro, 2004 [[116](#_ENREF_116)] | Not a systematic review |
| Salone, 2013 [[117](#_ENREF_117)] | Not a systematic review |
| Skinner, 2013 [[118](#_ENREF_118)] | Not a systematic review |
| Steyn, 2012 [[119](#_ENREF_119)] | Update of guidelines |
| Tanzer, 2001 [[120](#_ENREF_120)] | Systematic review on etiology |
| Theodoratou, 2014 [[121](#_ENREF_121)] | Review of reviews |
| Treasure, 2002 [[122](#_ENREF_122)] | Not a systematic review |
| Weyant, 2013 [[123](#_ENREF_123)] | Update of guidelines |

Table S3 legend. Excluded systematic reviews and the main reason for exclusion.
